# The potential for phenological mismatch between a perennial herb and its ground-nesting bee pollinator

**DOI:** 10.1093/aobpla/ply040

**Published:** 2018-07-02

**Authors:** Rachael L Olliff-Yang, Michael R Mesler

**Affiliations:** 1 Department of Integrative Biology, University of California, Berkeley, CA, USA; 2 Department of Biology, Humboldt State University, Arcata, CA, USA

**Keywords:** Asynchrony, climate warming, coastal dunes, mutualism, phenology, pollination mismatch, solitary bee

## Abstract

Climate change may alter the timing of flowering and pollinator activity to differing degrees, resulting in phenological mismatches between mutualistic partners. Assessing the potential for such mismatches requires an understanding of the environmental factors that cue flowering and pollinator activity. Biological context is key to determining specific impacts of climate change, and therefore it is important to study mutualisms with pollinators of different nesting biologies. Our study focused on the phenology of two mutualists native to the coastal dunes of northwestern California: the silky beach pea (*Lathyrus littoralis*) and its main pollinator, the ground-nesting solitary silver bee (*Habropoda miserabilis*). We measured the current phenological overlap between the two species and took advantage of local fine-scale spatial variation in the timing of flowering and bee nesting activity to develop predictive models of flowering and flight period timing based on variation in soil temperature and moisture. Temperature best predicted both flowering and bee activity, although soil moisture influenced the timing as well. Comparison of linear regression slopes of phenology against temperature suggests that bee nesting time is more sensitive to differences in seasonal maximum temperatures, and may advance more rapidly than flowering with temperature increases. Although the current phenological overlap between the two species is high, this differential response to temperature could result in a decrease in overlap with climate warming. Our results highlight that nesting biology may be critical in determining impacts of climate change on pollination mutualisms, as ground-nesting bees may respond differently than other bee species. In addition, this work reveals the utility of studying bee species that nest in aggregations for understanding ground-nesting bee phenology.

## Teaser

Flowering time is shifting with climate change. Pollinators may respond differently to changes in temperature and moisture than the flowers they visit, resulting in a timing mismatch between species. In this study, we evaluate the timing of a perennial plant and its main pollinator, a solitary ground-nesting bee. The timing of the bee was recorded at the nesting sites, independent of flowering. We find that the timing for both species is strongly associated with temperature, but that the bee’s nesting time may shift faster with climate change, revealing the potential for a timing mismatch in these species.

## Introduction

In the past two decades, the potential asynchrony of plant flowering and pollinator flight seasons has become a concern due to climate change. As abiotic factors in the environment are altered, species-specific shifts in phenology may occur (Forrest and Thomson 2011). Phenological events have already responded to increases in temperatures, often resulting in advanced spring timing (Menzel *et al.* 2006; IPCC 2007; Bartomeus *et al.* 2011; CaraDonna *et al.* 2014). While phenologies of plants and pollinators have shifted together in some cases (Bartomeus *et al.* 2011), such synchrony may not be true in all situations. With further changes in climate, timing mismatches between plants and their pollinators may develop (Memmott *et al.* 2007; Bartomeus *et al.* 2011; Willmer 2011). Such mismatches are more likely when plants and their pollinators respond to different abiotic cues to time their activity (Willmer 2011; Rafferty *et al.* 2015). The most parsimonious expectation would be that plants and their pollinators have adapted to respond to similar cues, due to shared co-evolutionary history, and the natural annual variability in weather. However, even if plants and pollinators cue into the same abiotic factors, there is a potential for mismatch if they respond to changes in those factors differently (Forrest and Thomson 2011). Therefore, it is important to assess how plants and pollinators respond to phenological cues that could contribute to asynchrony under future climatic conditions.

Many stimuli are known to induce flowering, including photoperiod, precipitation, threshold temperatures and interactions with other species (Willmer 2011). The factors that cue bee emergence are less well understood, especially for solitary, ground-nesting species (Forrest and Thomson 2011). The timing of insect activity is often associated with temperature cues, in particular degree-day accumulation (Forrest and Thomson 2011), and in heterogeneous habitats, microclimatic gradients can affect the timing of emergence (Weiss *et al.* 1993). In contrast to cavity-nesting bees, which can nest in blocks of wood and easily be used in experiments (e.g. Fründ *et al.* 2013), the nests of ground-nesting (also called ‘mining’) bees are often difficult to find and challenging to work with experimentally. While there have been some studies on mining bee phenology cuing in a lab setting (e.g. Danforth 1999), and using cages (e.g. Schäffler and Dötterl 2011), almost no studies have been done without artificial manipulation of the nests or habitat (but see Minckley *et al.* 1994).

**Figure 1.**
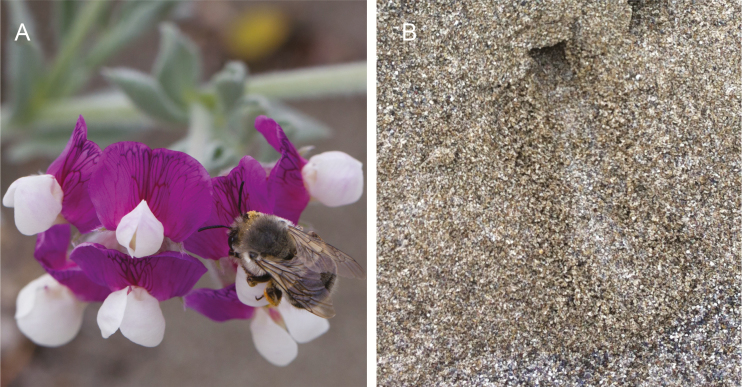
(A) *Habropoda miserabilis* (silver bee) female pollinating *Lathyrus littoralis* (beach pea); (B) active nest entrance of *H. miserabilis*. Note the pattern in front of entrance, consisting of parallel bands of sand pushed to the side during digging. Zigzag marks across the path are also characteristic (not shown).

Our study focused on the phenology of two mutualists native to the coastal dunes of Humboldt County, CA: the silky beach pea (*Lathyrus littoralis*) and its main pollinator, the ground-nesting solitary silver bee (*Habropoda miserabilis*) (Fig. 1A). There is significant spatial variation in the phenology of both *L. littoralis* and *H. miserabilis* (Olliff 2014). *Lathyrus littoralis* has been observed to bloom later in the season in the northern areas of Humboldt Bay (Monroe 2010), and in cooler landscape positions (e.g. north-facing slopes; Olliff 2014). Likewise, *H. miserabilis* exhibits distinct variability in flight timing. Differences in the timing of nesting have been reported along a north–south gradient, sometimes beginning and ending almost a month earlier in the southern end of its range in Humboldt County (Monroe 2010). This spatial variation in both flower and bee phenology allowed us to develop models to evaluate potential abiotic cues for the timing of these species.

Studying the phenology of plants and pollinators separately allows for the detection of unique cues for each (Forrest and Thomson 2011). Mining bees that nest in aggregations offer an opportunity to measure the phenology of these types of bees at their nest site. *Habropoda miserabilis* create obvious nesting aggregations in open sand dunes. These aggregations are stable and can persist for many years. The open nature of the sand dunes also makes it feasible to find and quantify the number of active nests. Because of this nesting strategy, we were able to document the phenology of this species at its nesting sites.

In this study, we tested whether variation in phenology is correlated with intra-annual variation in temperature and moisture. Specifically, we used the fine-scale, local differences in abiotic variables as a proxy for variation in climate over time (Forrest and Thomson 2011), and in lieu of experimental manipulation of abiotic variables. The objectives of this study were to (i) quantify existing overlap of the flight season of *H. miserabilis* and flowering time of *L. littoralis*, (ii) test the responses of bee nesting activity and blooming to two likely phenological cues—temperature and moisture.

## Methods

We conducted this study in northwestern California, along the North Spit of Humboldt Bay (40°50′28.33″N, 124°06′11.92″W), in 2013. This area has a mild Mediterranean-type climate, with most precipitation received in the winter, and warmer temperatures in the summer. Temperature is buffered year round by the proximity to the ocean, with an average temperature (during 1986–2014) of 10.8 °C (average yearly max: 14.6 °C, average yearly min: 7.1 °C; NOAA 2014). Five sites were chosen for monitoring nesting aggregations of *H. miserabilis.* Flowering of *L. littoralis* was also monitored at five sites, but this species does not occur across the full range of the North Spit, and was not present at the two southernmost sites chosen for bee monitoring. To account for this, two additional nearby flower sites were included and compared to the southern bee sites **[see****Supporting Information—Fig. S1****]**. These two southern sites were within the estimated foraging distance of the southern bee sites (est. 3000 m; Monroe 2010).

*Lathyrus littoralis*, or silky beach pea, is a native legume that plays an important role in the establishment and early succession of dune vegetation in the coastal dunes of Northern California (Pickart and Sawyer 1998). *Lathyrus littoralis* is one of the first plants on the dunes to bloom in the spring and likely an important early floral resource for pollinators (Gordon 1984). This species requires pollinator visits for seed set (Olliff 2014). *Habropoda miserabilis* (dune silver bee) is one of the most common bee species in the dunes of Humboldt Bay. It is a generalist bee species, and nests in persistent aggregations in the open sand where nests can easily be counted. It is the most frequent visitor of *L. littoralis*, and provided 58 % of the pollinator visitation observed during this study (ranged from 33 to 87 % of visits at each site; **see****Supporting Information—Fig. S2**; Olliff 2014). *Habropoda miserabilis* is likely the most important pollinator of *L. littoralis* (Gordon 1984). Both are early spring species, and are therefore predicted to be phenologically responsive to changes in climate (Petanidou *et al.* 1995; Bartomeus *et al.* 2011).

### Measurement of flowering and nesting phenology

To measure the flowering phenology of *L. littoralis*, we sampled 30 circular 3-m-diameter plots across five sites in 2013 (6 plots per site). The vegetation at each site was characterized by seral stage, representing different levels of succession since dune movement, from early (including *L. littoralis* and no other plant species), mid (including *L. littoralis* and other species but <10 % Poaceae) and late (including *L. littoralis* and other species and >10 % Poaceae) stages. Plots were chosen randomly from existing patches of *L. littoralis*, with the constraint that 2 plots per site were selected from within each seral stage class to account for potential effects of competition and shading differences on phenology.

In lieu of counting all open flowers per plot, the number of inflorescences with at least 50 % open flowers was counted on average every 6 days (±1.5 days SD) (Norris 1996; Totland *et al.* 2006; Fava *et al.* 2011). Inflorescences were not counted after >50 % of flowers had senesced and were no longer attractive to pollinators. Senescence was determined by a change in banner petal colour (fading from bright pink-purple to a dull blue), and keel petal color (bright white to white-brown). From these counts we determined the start, peak (highest number) and end dates of flowering.

To characterize the nesting season of *H. miserabilis*, we observed activity at 17 aggregations across five sites, chosen randomly from a pool of 41 aggregations documented in previous years. We chose three nesting aggregations (‘bee plots’ hereafter) at three sites (LAN, SA, EPA) and four in two sites with more frequent disturbance (due to high human foot traffic and recreation): (MR and MA), and monitored them on average every 6 days (±1.5 days SD), varying the order of the plot visits on each monitoring day. Bee plots varied from 18 to 1372 total nests over the entire season, and ranged from ~10 to 100 m^2^**[see****Supporting Information—Table S1****]**.

On each census date, we counted the number of active nests in each plot. Counting active nests entailed temporarily marking nest entrances where female activity was seen (digging, entering and exiting nests), as well as where fresh digging tracks were observed. Females dig to build their nests by pushing sand backwards, leaving distinctive patterns at the nest entrance (Fig. 1B; **see****Supporting Information—Video**). These patterns disappear quickly (typically within 1–24 h after bee activity, depending on wind conditions), and are a good indication of recent activity. We spent at least 2 h at each plot, and if any new nests were found within the last 5 min of this monitoring period, the interval was extended by 30 min. We chose a 2-h monitoring period based on the longest recorded foraging trips for *H. miserabilis* (Monroe 2010).

From these counts we determined the start, peak and end dates of bee nesting. Start and end dates were the first and last dates, respectively, of recorded active nests. Peak nesting was defined as the date with the highest abundance of active nests present, as the distribution of nesting across time is unimodal. Abiotic variables are expected to directly impact emergence from diapause, and indirectly influence nesting times. We measured nesting time as a proxy for emergence timing. Although bees emerged from diapause a few days prior to the start of nesting, the number of active nests present at a given time reflects the pattern of emergence across the season. Nesting bees are also actively collecting pollen and nectar, and this timing therefore coincides with the most pollination services to the flowers.

**Figure 2.**
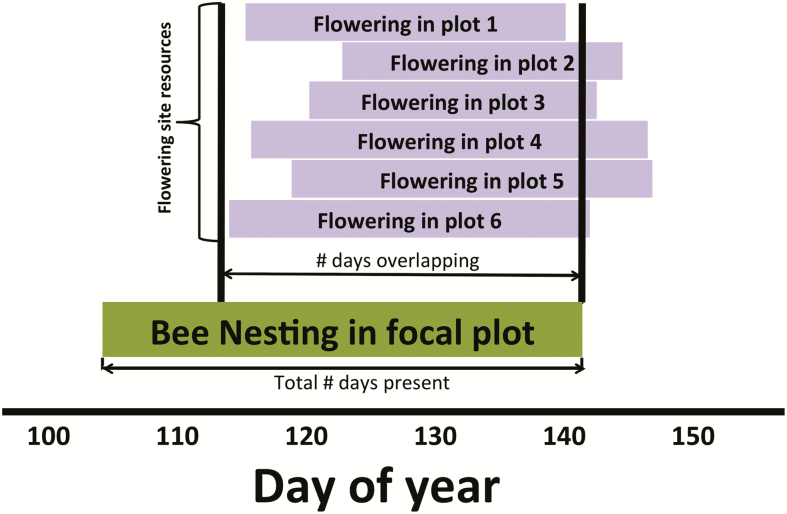
Schematic diagram demonstrating the percentage of days overlap calculation. Bars indicate the dates over which the species in the focal plot is active (in this case bees, dark green bar), and the site resources were present (in this case flowers, light purple bars). All ‘resource’ plots at a site were pooled to determine each plot’s overlap with site resources. Overlap for each plot was calculated as: (Total # of days overlapping/# number of days present) * 100.

Percentage of overlapping days for flowering and bee nesting was then calculated. This was defined as the percentage of days of overlap between each plot and the site resources (Fig. 2). Specifically, the overlap of a bee plot was the percentage of days the plot had active nests and there were also flowers present at any of the flower plots in that site (and vice versa for flowers and pollinator resources). The calculation used was:

$${\text{Percent Overlap}}_{i}=\frac{\#{\text{ days overlapping}}_{j}}{\text{total }\#{\text{ days present}}_{i}}\;*100$$

where *i* is the focal species at a plot (bees or flowers), and *j* is the site resources (flowers present or nesting bees, respectively).

Asynchrony was also calculated, in order to incorporate the full phenology distribution of both species. Asynchrony was defined as the difference in the mean phenological dates between the plots of the focal species and the surrounding site resources (Donoso *et al.* 2016). The ‘mean date’ was calculated in three steps (as in Donoso *et al.* 2016): (i) computing the mean of the first and last dates, (ii) finding the date at which 50 % activity was reached and finally (iii) taking the mean of both dates in steps 1 and 2. The mean date for each plot was then subtracted from the mean site date, and the absolute value of this difference was the asynchrony. The calculations were:

$$\text{Mean Date}=\frac{\frac{fir\text{st DOY}+\text{last DOY}}{2}+\text{DOY }50\text{\% activity }}{2}$$

and

$${\text{Asynchrony}}_{i}=|{\text{Mean DOY}}_{i}-{\text{Mean DOY}}_{j}|$$

where DOY stands for ‘day of year’, *i* is the focal species at a plot (bees or flowers), and *j* is the site resources (flowers present or nesting bees, respectively).

### Potential cues for flowering and bee emergence

Air temperatures and rainfall are similar across all sites given their proximity, but we expected differences in topography to cause variation in soil temperature and moisture that could influence the timing of bee nesting and flowering. Topography can create substantial variation in temperature and moisture across fine spatial scales (Geiger *et al.* 2009; Oldfather *et al.* 2016). To test this, soil temperature and moisture measurements were made every 6 days (±1.5 days SD) starting in February and ending in May. These measurements were taken on the same days we counted flowers and nests. Soil temperature was measured in each plot at both 1 and 25 cm depths below the surface with a Fluke 5II Thermometer probe. These depths were chosen to represent a near-surface point (1 cm) and the shallowest depth where sand-nesting *Habropoda* bees in diapause are found (25 cm; Barthell *et al.* 1998). Soil moisture (volumetric water content) was measured just below the surface with a Decagon 5TE Soil Moisture Sensor (Decagon Devices 2014). These variables were measured at the centre of flower plots, and averaged from multiple points in bee plots (taken at stratified points at the same time [±30 min], *n* = 3–7 points depending on plot size). Overall season minimum, maximum, and average temperature and moisture from February through May were calculated from these measurements. Degree-day accumulation was not calculated, as temperatures were not taken continuously. However, higher temperature plots can be expected to have faster degree-day accumulation. One limitation of this study is that temperatures before February were not recorded. Temperatures from earlier in the year may affect bee development and emergence timing as well as the timing of flowering, but this was not tested in this study.

Because populations within each species studied were relatively close together (max distance between populations < 20 km), genetic differentiation for cuing mechanisms was not expected. Therefore, a correlation of observed activity dates with abiotic variables measured over a set period of time would indicate the potential cuing mechanisms. For example, a plant species triggered to flower after a threshold number of accumulated temperature degree-days is reached, will reach this threshold faster in warmer sites (e.g. Iler *et al.* 2013). Similarly, cooler plots can be expected to reach any chilling requirement faster and more completely.

We used linear mixed models to determine if the start, peak and end dates of phenological events were related to soil temperature, soil moisture or an interaction of the two. Regressions were performed with site as a random variable, specified as a random intercept with a fixed mean (R package lme4; Bates *et al.* 2015). To assess the best abiotic predictor variable, models were compared using AICc values (R package AICcmodavg; Mazerolle 2017). Once the best predictor variables were identified, AICc values were used to assess whether additional variables and an interaction of temperature and soil moisture were significant predictors of the phenological events. Model fits were assessed using marginal *R*^2^ values and conditional *R*^2^ values (function r.squared GLMM in R package MuMIn; Bartoń 2016). All analyses in this study were performed in R (version 3.3.1; R Core Team 2016).

A critical assumption of this study is that bees nesting at the plots had emerged from the same area, and experienced the abiotic conditions in that plot prior to emergence and nesting activity. Although we did not verify this assumption, philopatry has been reported for other solitary bees that nest in aggregations (Sakagami and Michener 1962; Yanega 1990; Potts and Willmer 1997; Soucy 2002), and is consistent with the long-term stability of nest aggregations in the study area.

## Results

### Measurement of flowering and nesting phenology

**Figure 3.**
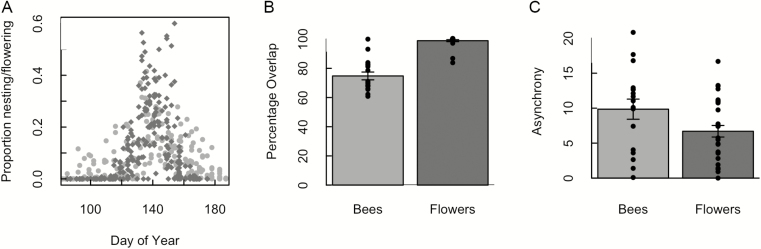
Differences in (A and B) overlap and (C) asynchrony of flowering and nesting phenology plots at each site (*n* = 17 bee plots, *n* = 30 flower plots). Proportion activity is the proportion of total season flowering (dark grey diamonds) and nesting (light grey circles) at each plot on a given day [calculated as # bee nest or inflorescences present on date/# present over total season] (A). Overlap (B) is the percentage of days where bee nesting at a plot occurred with flowering resources at a site (and vice versa for flowers). Asynchrony (C) is the difference in mean dates between bee nesting at a plot and flowering resources at a site (and vice versa for flowers), calculated as in Donoso *et al.* (2016). Raw overlap (B) and asynchrony (C) values are overlaid as points on the bar graphs. Error bars show 1 SEM. See methods text for calculations.

Nesting and flowering occurred between March and July, and overlapped strongly. The start of bee activity and flowering varied from late March to early May, and stopped between mid-June to early July (Fig. 3A). The blooming period overlapped 74.7 % (±0.11 % SD, *n* = 17) with bee nesting, and bee nesting overlapped 98.9 % (±0.037 % SD, *n* = 30) with blooming overall (Fig. 3A and B). The degree of asynchrony with the surrounding site resources was higher for bees than flowers, with a mean of 9.32 (±5.4 days SD, *n* = 17) for the bees and 7.07 (±4.3 days SD, *n* = 30) for the flowers (Fig. 3C).

### Potential abiotic cues for flowering and bee emergence

**Figure 4.**
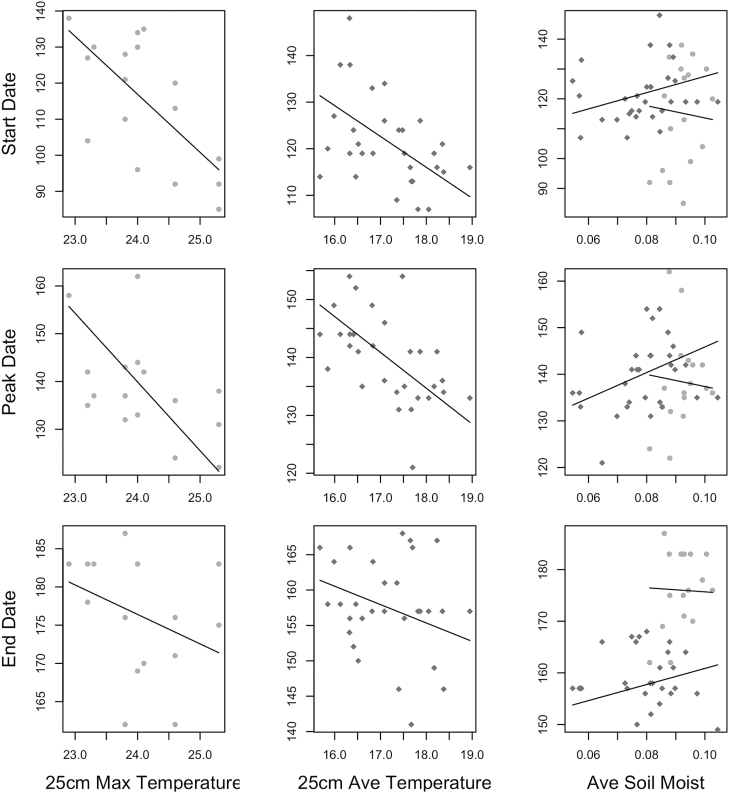
Start, peak and end dates (day of year) of silver bee nesting (light grey circles) and beach pea flowering (dark grey diamonds) in relation to average soil temperatures (°C) at 25 cm (best predictor for flowering), maximum temperatures (°C) at 25 cm (best predictor for nesting) and average soil moisture (VWC, included in all best-fit models). Slope and intercepts for the lines were plotted from the fixed effects of the best-fit mixed models (scaled to the mean value of the other fixed effect in the model). Effect size and direction of fixed factors are included in Table 2.

The timing of both bee activity and flowering responded to soil temperature and moisture, but in different ways. Model comparison revealed that temperature measured at 25 cm below ground was the best predictor of both nesting activity and flowering dates (Tables 1 and 2), with temperature negatively related to timing (Table 2). In other words, sites with warmer temperatures exhibited earlier phenology. This relationship was especially strong for start and peak timing (Table 2). Soil moisture, and the interaction of moisture and temperature, also contributed to timing (Table 2); however, the direction of the slopes for soil moisture effects was opposite for bees and flowers (Fig. 4; Table 2; for interaction contour plots—**see****Supporting Information—Fig. S3**). The best models explained more variance in bee nesting dates than for flowering dates (higher *R*^2^ values; Table 2).

**Table 1.** Mixed effect linear models of bee nesting and flowering phenology in response to climate variables: temperature (*T*) and soil moisture (*M*). Temperatures were measured at both 1 cm (1) and 25 cm (25) below the surface of the soil. The bold rows indicate which models performed best based on AICc comparisons. Flowering end dates were equally well predicted by 1 and 25 cm average below-ground temperatures, as there was <1 AICc unit difference.

Bee nesting				Flowering			
Phenology	Model	AICc	∆AICc	Phenology	Model	AICc	∆AICc
Start	**25 *T***_**max**_	**3046.1**	**0**	Start	**25 *T***_**ave**_	**2637.2**	**0**
	25 *T*_min_	3084.4	38.3		1 *T*_max_	2642.1	4.8
	1 *T*_max_	3183.1	136.9		1 *T*_min_	2679.5	42.3
	1 *T*_min_	3201.5	155.5		25 *T*_max_	2683.5	46.3
	Null	3213.6	167.4		25 *T*_min_	2683.8	46.5
	*M*_ave_	3213.9	167.9		1 *T*_ave_	2683.9	46.7
	1 *T*_ave_	3214.7	168.6		*M*_ave_	2690.1	52.9
	25 *T*_ave_	3215.0	168.9		Null	2695.4	58.1
Peak	**25 *T***_**max**_	**2711.1**	**0**	Peak	**25 *T***_**ave**_	**2391.7**	**0**
	25 *T*_min_	2891.5	180.4		25 *T*_min_	2415.8	24.2
	1 *T*_max_	2906.7	195.6		1 *T*_ave_	2435.3	43.6
	25 *T*_ave_	2937.8	226.7		1 *T*_max_	2460.7	69.1
	1 *T*_ave_	2944.4	233.3		25 *T*_max_	2473.7	82.0
	1 *T*_min_	2962.9	251.9		*M*_ave_	2487.9	96.2
	Null	2973.8	262.7		Null	2490.0	98.3
	*M*_ave_	2975.4	264.3		1 *T*_min_	2491.1	99.4
End	**25 *T***_**max**_	**2127.7**	**0**	End	**1 *T***_**ave**_	**2433.6**	**0**
	1 *T*_ave_	2171.3	43.6		**25 *T***_**ave**_	**2433.9**	**0.3**
	1 *T*_min_	2180.8	53.1		25 *T*_min_	2438.5	4.9
	1 *T*_max_	2208.7	80.9		1 *T*_max_	2442.8	9.2
	25 *T*_ave_	2211.3	83.6		1 *T*_min_	2451.8	18.2
	Null	2221.8	94.1		Null	2451.9	18.3
	25 *T*_min_	2221.9	94.2		25 *T*_max_	2452.2	18.5
	*M*_ave_	2223.0	95.3		*M*_ave_	2453.9	20.3

**Table 2.** Mixed effect linear models of bee nesting and flowering phenology in response to climate variables: temperature (*T*) and soil moisture (*M*). Temperatures used were from best-fit models based on AICc comparison (Table 1), and were measured at both 1 cm (1) and 25 cm (25) below the surface of the soil. The bold AICc values indicate which models performed best based on AICc comparisons. *R*^2^m indicates marginal *R*^2^ values, and *R*^2^c indicates conditional *R*^2^ values of the models. Slope estimates for all variables as well as the SE of the estimates are shown for all models, and the estimates of the best models are highlighted in bold font.

Bee nesting						Flowering					
Phenology	Model	AICc	*R* ^2^m	*R* ^2^c	Slope est. ± SE	Phenology	Model	AICc	*R* ^2^m	*R* ^2^c	Slope est. ± SE
Start	**25 *T***_**max**_	3046.1	0.444	0.685	−15.27 ± 1.05	Start	**25 *T***_**ave**_	2683.9	0.227	0.307	−5.4647 ± 0.7
	*T* + ***M***	3044.2	0.420	0.702	−15.34 ± 1.05 −240.5 ± 118.7		*T* + ***M***	**2612.3**	**0.367**	**0.510**	−**6.62 ± 0.69** **272.7 ± 50.1**
	*T* + *M* + ***T:M***	**2975.3**	**0.484**	**0.728**	−**132.2 ± 13.31** −**30536 ± 3445** **1258.3 ± 143**		*T* + *M* + ***T:M***	2612.8	0.372	0.519	−1.466 ± 4.219 1417 ± 924 −64.8 ± 52.3
Peak	**25 *T***_**max**_	2711.1	0.524	0.796	−13.66 ± 0.7	Peak	**25 *T***_**ave**_	2391.7	0.337	0.438	−5.301 ± 0.49
	*T* + ***M***	2709.1	0.515	0.7997	−13.72 ± 0.7 −159.87 ± 78.9		*T* + ***M***	2363.4	0.461	0.599	−6.1487 ± 0.5 205.4 ± 36.04
	*T* + *M* + ***T:M***	**2581.8**	**0.584**	**0.844**	−**114.9 ± 8.2** −**26380 ± 2127** **1088.99 ± 88**		*T* + *M* + ***T:M***	**2354.6**	**0.477**	**0.625**	**3.6 ± 2.98** **2374.7 ± 652.7** −**122.8 ± 36.9**
End	**25 *T***_**max**_	2127.7	0.124	0.832	−3.586 ± 0.3	End	**1 *T***_**ave**_	2433.6	0.086	0.198	−1.369 ± 0.3
	*T* + ***M***	2128.0	0.120	0.837	−3.60 ± 0.34 −51.8 ± 38.79		*T* + ***M***	2435.3	0.080	0.183	−1.386 ± 0.3 24.2 ± 37.6
	*T* + *M* + ***T:M***	**2049.5**	**0.146**	**0.863**	−**43.8 ± 4.27** −**10487 ± 1106** **433.4 ± 45.9**		*T* + *M* + ***T:M***	**2416.2**	**0.128**	**0.227**	**5.98 ± 1.61** **1996 ± 424.4** −**91.2 ± 19.55**

 Flowering start, peak and end dates responded similarly to both average and maximum temperature—with shallow negative slopes (Fig. 4; Table 2). Plots with warmer average temperatures exhibited earlier flowering, by ~6.6 days per 1 °C increase in average temperature (start date regression slope—6.6; Table 2). The timing of bee nesting was more responsive to maximum temperatures than average temperatures at 25 cm below the soil surface. Plots with warmer maximum temperatures exhibited earlier nesting behaviour by ~15.3 days per 1 °C increase (start date regression slope—15.3; Table 2). The response in nesting time to increase in maximum temperatures was much greater in magnitude than the flowering time response to average temperature increase, as shown by the steeper regression slopes (Fig. 4; Table 2). However, maximum temperature was not a good predictor of flowering time (Table 1; marginal *R*^2^ = 0.04, 0.05 and 0.006 for start, peak and end dates, respectively), and average temperature was not a good predictor of bee nesting (Table 1; marginal *R*^2^ = 0.002, 0.13 and 0.016 for start, peak and end dates, respectively), so the slopes of the best-fit models for the bees and flowers cannot be directly compared.

## Discussion

There is a growing concern that increases in global temperatures may cause phenological mismatches between flowering plants and their pollinators. Some studies have shown that flowers and their pollinators seem to be keeping pace with one another (Bartomeus *et al.* 2011; Iler *et al.* 2013), while others suggest increasing asynchrony as warming continues (McKinney *et al.* 2012). Due to the fast pace of climate change, it is important to assess the cuing mechanisms of characteristic mutualist species to determine the potential for asynchrony in a variety of systems. This study presents a pair of species that both respond to temperature cues but react to different aspects of temperature. Bee nesting time was especially responsive to maximum temperatures, while flowering time was more closely tied to average temperatures. This suggests that an increase in extreme heat events might increase asynchrony in the future.

Studies conducted in other Mediterranean-type climate areas have also shown that temperatures (Petanidou *et al.* 1995) and precipitation (Mazer *et al.* 2015) in months preceding flowering are important. Climate seems to be a significant driver of plant–pollinator interactions in these systems (Petanidou *et al.* 2017), and timing shifts in early flowering species have been shown to decrease pollination services in some cases (Petanidou *et al.* 2014). While similar relationships to temperature and moisture are shown here, this study further elucidates how a pair of mutualistic species might respond differently to changes in these factors by examining the timing of a plant and its main pollinator separately.

Our models suggest that both the timing of nesting and flowering may advance in warmer years (Table 2; Fig. 4). However, an increase in maximum temperatures is predicted to have a larger impact on the timing of *H. miserabilis*, while average temperatures are more influential for flowering time (Table 1). The difference in slopes of the best-fit models for each species suggests that the magnitude of response to a temperature increase could yield a faster advance in the timing of bee nesting than flowering time (Fig. 4; Table 2). Flowering peak time is predicted advance ~5.2 days per 1 °C increase in average temperature at 25 cm soil depth (scaled temperature marginal regression slope: −5.2; Fig. 4), while peak bee nesting is predicted to advance by ~10.6 days per 1 °C increase in maximum temperatures at 25 cm soil depth (scaled temperature marginal regression slope: −10.6; Fig. 4). Bee nesting time was especially responsive to maximum temperatures, so an increase in extreme heat events, even if average temperatures are unchanged, may reduce overlap between these species. However, it is difficult to predict exactly how increased temperatures will shift nesting and flowering timing, as the temperature measures for the best-fit models for the bees and flowers are different (maximum vs. average temperatures), and their slopes cannot be directly compared.

The interaction of temperature with soil moisture also contributed to differences in timing cues, as the slopes of soil moisture were opposite in best-fit bee versus flower models (Table 2; Fig. 4). The effect of temperature on nesting and flowering time is dependent on soil moisture (Table 2). Increased moisture was positively related to flowering time, indicating a delay in flowering dates in wetter plots. Bee nesting on the other hand had a shallow negative relationship with soil moisture. This opposite response to soil moisture reveals that flowering time is delayed with increased soil moisture, while bee nesting time advances in wetter plots. However, the effect of soil moisture on timing depends on soil temperatures (see interaction contour plots—**Supporting Information—Fig. S3**).

Interactions of temperature with day length cues may also moderate timing cues in *L. littoralis*. Though this study could not test day length cues, the best-fit models for flowering explained a smaller portion of the variation in flowering dates than bee nesting dates (Table 2), revealing that the timing of *L. littoralis* flowering may be less dependent on temperature and moisture cues. Plant reproductive cycles in temperate zones are often cued by a combination of day length and temperature (Menzel 2002). If day length is an important flowering cue for *L. littoralis*, it would moderate the dependence on temperature as a cue for blooming by constraining flowering to a set of similar dates each year.

Our results suggest a faster shift in bee than flowering phenology with spring temperature increases, in contrast to similar studies on cavity-nesting bees (e.g. Forrest *et al.* 2011). Soil likely buffers temperatures experienced by *H. miserabilis*, but paradoxically bee nesting time was more sensitive to temperature increases than flowering time (Fig. 4; Tables 1 and 2). This finding may have implications for other ground-nesting bee species, in contrast to cavity-nesting species. Solitary cavity-nesting bees tend to overwinter in substrate above ground (e.g. dead wood and pithy stems), and spring temperatures are not buffered by the soil, thus subject to greater extremes. Where and how bees are experiencing temperatures may result in differential responses to warming. While this would rely heavily on the specific conditions of each system, this distinction may be key, as ground-nesting bees make up ~70 % of native bee species in the USA (Shepherd 2012). This result highlights the importance of context and biology in determining the impacts of climate change.

The effect of pollination mismatch on demography and evolutionary trajectories of bee and flowering plant populations has largely been understudied. Only a few cases have documented an impact on population vital rates, and these were mainly through a reduction in seed production (Forrest 2015). Shifts in this system might not have much of an effect on *L. littoralis* seed set, as reduction in overlap with *H. miserabilis* might predominantly occur at the end of the season when bumblebees are more common. In contrast, *H. miserabilis* may be more affected by any projected shift, as its early spring timing may shift faster than *L. littoralis*, as this species is often the earliest floral resource available and bees depend on floral resources for successful reproduction. Male silver bees might be especially impacted, as they exhibit protandry and are active earlier than the nesting females. If floral resources are not available early in the season, any early bees would not be able to survive. While this is a concern, it would lead to a strong selection for later season bees, and not necessarily result in a long-term decline in the population (Altermatt 2010). Close monitoring at the nest site will be necessary to determine if bees are emerging increasingly earlier than the flowers in the spring, and whether any declines occur due to phenological shifts.

There are currently very few suggestions for managing a system for climate-induced asynchrony. Ensuring that plants are established on a variety of slopes and aspects may help mitigate some problems due to shifting phenology, as temperature and moisture will differ depending on these factors. It will also be important to continue the ongoing restoration practices on the dunes, as removing invasive species allows for the persistence and diversity of native flowering plants and suitable *H. miserabilis* nesting habitat.

## Conclusions

This study showed that the overlap of two mutualistic species, the perennial plant *L. littoralis* and the ground-nesting bee *H. miserabilis*, is currently high, but that there is a difference in how these species respond to both temperature and moisture cues. For this reason, an increase in temperatures, especially extreme heat events, could shift the timing of these species apart in the future.

Phenology overlap data generated through this study can be used as a baseline to refer to when monitoring changes over time, and to assess future synchrony of this pollination mutualism. These data were collected over 1 year, and in a limited number of sites across only a portion of the species’ ranges, but phenological monitoring of both species is ongoing via the National Phenology Network and California Phenology Project citizen science programmes (Haggerty *et al.* 2013). In addition, the cuing mechanisms in these two species may be similar to other species, and provide greater understanding of solitary ground-nesting bee phenology. Insights from this study can be used to inform the conservation of these two species, and may influence management considerations of similar species.

## Sources of Funding

This research was funded by a Conservation Research Grant from the Sequoia Park Zoo (Eureka, CA, USA), a Northern California Botanists Research Scholarship (USA), a Richard J. Guadagno Memorial Scholarship from the Fish and Wildlife Service (California, USA), a Humboldt State University Graduate Student Research Grant (California, USA) and a Gary J. Brusca Zoology Scholarship (USA). Publication made possible in part by support from the Berkeley Research Impact Initiative (BRII) sponsored by the UC Berkeley Library. Additional support was provided by the National Science Foundation Graduate Research Fellowship Grant (#1049702) (to R.L.O.-Y.). Any opinion, findings, and conclusions or recommendations expressed in this material are those of the authors and do not necessarily reflect the views of the National Science Foundation.

## Contributions by the Authors

R.L.O.-Y. conceived and designed the research, collected and analysed the data, and wrote the manuscript. M.R.M. advised the project and revised the manuscript. Code and data available: https://github.com/rlolliff/Silverbee.

## Conflict of Interest

None declared.

## Supplementary Material

Supplementary Video

Supplementary Information

## Supporting Information

The following additional information is available in the online version of this article—

**Video.** Female silver bee (*Habropoda miserabilis*) digging a nest.

**Figure S1.** Map of site locations along the North Spit of Humboldt Bay.

**Figure S2.** Relative abundance of visitors to *Lathyrus littoralis* flowers.

**Figure S3.** Soil temperature × moisture interaction contour plot.

**Table S1.** Location and size of bee aggregations (bee plots).
