# Supplementary material for: The potential for phenological mismatch between a perennial herb and its ground-nesting bee pollinator
Source: AoB Plants. 2018 Jul 2;10(4):ply040. doi: 10.1093/aobpla/ply040 (PMC6054160; doi:10.1093/aobpla/ply040)
Supplement: Supplementary Information [file ply040_suppl_supporting_information.pdf]

SUPPORTING INFORMATION

for

The potential for phenological mismatch between a perennial herb and its ground nesting bee pollinator

Authors:

R. L. Olliff-Yang and M. R. Mesler

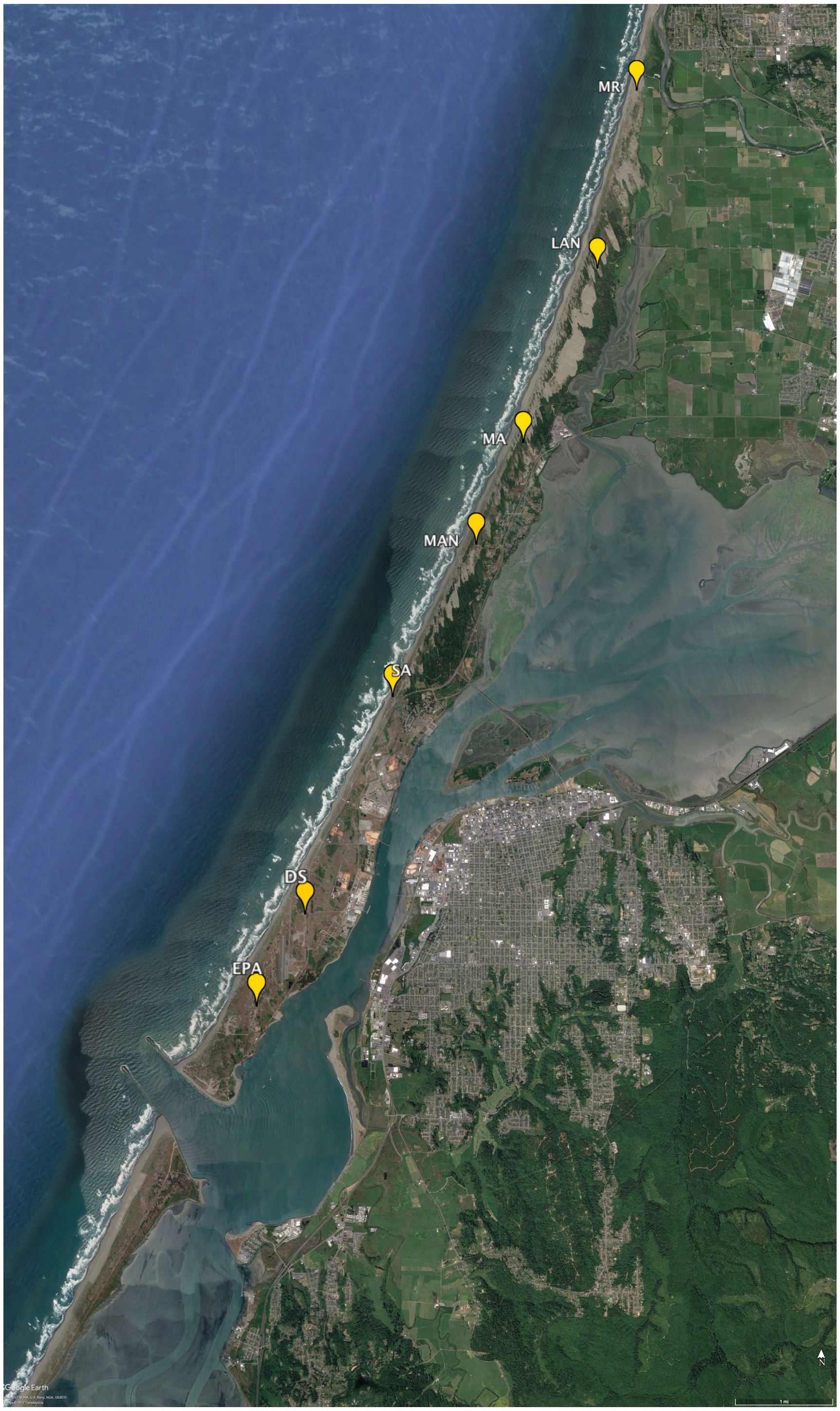

Figure S1 - Site locations along the North Spit of Humboldt Bay.

Sites with bee plots include Mad River Beach (MR, 4 plots), the Lanphere Dunes Unit of the Humboldt Bay National Wildlife Refuge (LAN, 3 aggregations), Ma-l'el Dunes South (MA, 4 plots), The Samoa Drag Strip Area (DS, 3 plots), and the Eureka Protected Area

(EPA, 3 plots). Sites with flower plots include MR, LAN, MA, Manila Dunes (MAN) and Samoa Beach (SA). All sites with *L. littoralis* included 6 plots. Imagery from Google Earth Google Earth™ (Google Inc. 2013).

Citations

Google Inc. 2013. Google Earth (Version 7.1.2.2041) [Computer program]. Available at: http://www.google.com/earth/download/ge/. (Accessed 29 December 2017).

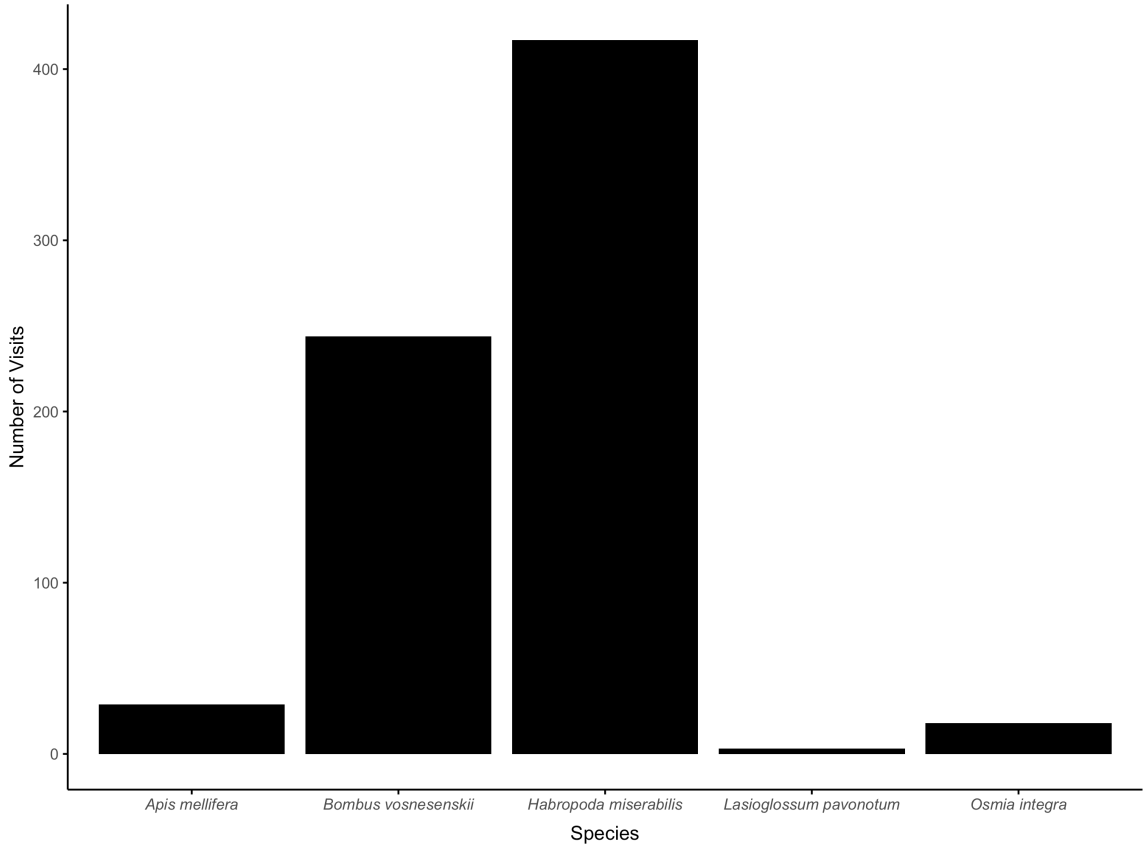

Figure S2 - Relative abundance of visitors to *Lathyrus littoralis* flowers. Visits to *L. littoralis* were observed to determine the relative abundances of different pollinators. Every time plots were visited to count flowers, the type of pollinator and number visits to flowers in each plot was recorded over a 10-minute interval. Over the blooming period we performed 180 pollinator observation periods, cumulatively totaling 1,800 minutes of observation time. All observation periods occurred between 11am and 3pm, and the order of plots visited was randomized each observation day. The number of flower visits by each species was recorded, and pooled across sites. 711 total visits were observed, and *Habropoda miserabilis* conducted 58.6% of these visits (n=417 of 711 visits). The percentage of visits by site: MR: 87.3% (n = 138 of 158 visits), LAN: 52.4% (n = 150 of 286 visits), MA: 50.2% (n = 120 of 239) MAN: 50.0% (n= 2 of 4 visits) and SA: 33.3% (n = 7 of 21 visits).

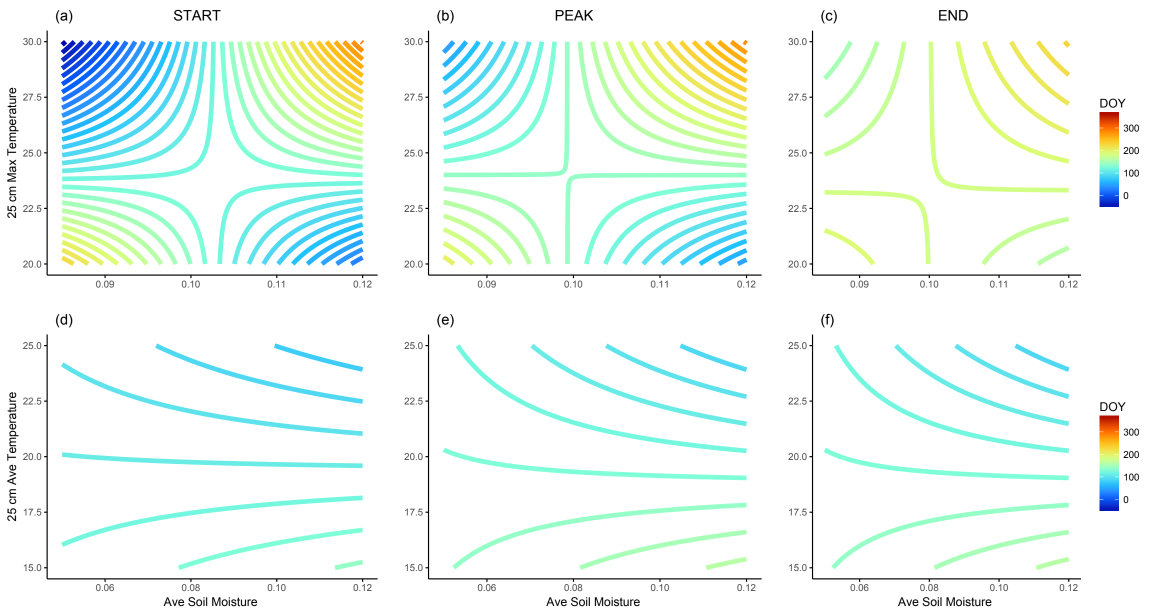

Figure S3 - Soil temperature x moisture interaction contour plot. Start, peak and end dates (Day of Year [DOY]) of silver bee nesting (a-c) and beach pea flowering (d-f) in relation to maximum soil temperatures (˚C) at 25 cm depth (a-c, best predictor for nesting dates), average soil temperatures (˚C) at 25 cm depth (d-f, best predictor for flowering dates), and average soil moisture (VWC fraction; included in all best fit models). Contour bin width is 10 days for all plots. Random effects were excluded from the models to draw fixed effect contours.

Table S1

Location and size of bee aggregations (bee plots). Location information is in WIGS 84 UTM. Start refers to the first date bees were seen active at the aggregation, and end is the last bee activity seen at the aggregation (date of last active nest) in 2013. Size refers to the total number of nests counted over the entire season in 2013. Aggregation size was not significantly related to bee phenology dates.

Site	Plot	Type	Zone	Easting	Northing	Start	End	Size
MR	1	Bee	10T	0404533	4531478	5/1	7/2	257
MR	2	Bee	10T	0404442	4531273	5/4	7/2	346
MR	3	Bee	10T	0404412	4531203	5/1	7/2	23
MR	4	Bee	10T	0404377	4531323	4/24	7/2	590
MA	1	Bee	10T	0402165	4523984	3/26	6/24	1372
MA	2	Bee	10T	0402057	4524519	5/2	7/2	135
MA	3	Bee	10T	0402161	4524454	4/9	7/2	353
MA	4	Bee	10T	0402193	4524234	4/2	6/17	172
DS	1	Bee	10T	0398066	4515373	4/23	6/20	480
DS	2	Bee	10T	0398068	4515386	4/23	6/20	962
DS	3	Bee	10T	0398091	4515595	4/30	6/25	324
EPA	1	Bee	10T	0397480	4514470	4/6	6/18	873
EPA	2	Bee	10T	0397278	4514308	4/20	6/11	197
EPA	3	Bee	10T	0397257	4514292	4/6	6/11	418
LAN	1	Bee	10T	0403380	4527271	4/14	6/27	270
LAN	2	Bee	10T	0403526	4527701	4/29	7/2	1113
LAN	3	Bee	10T	0403572	4527639	5/7	6/27	18
